# Hospital and Institutional News

**Published:** 1914-06-06

**Authors:** 


### HOSPITAL AND INSTITUTIONAL NEWS.

### THE ULSTER HOSPITAL CORPS' APPEAL.

The medical service of the counties Derry and Donegal, and of Captain Craig's hospital at Downpatrick, has been undertaken by the Ulster Hospital Corps, working in association with the medical authorities at Belfast. The Ulster Hospital Corps has therefore issued an appeal for surgeons and nurses who are willing to volunteer in the event of civil war in Ulster. Volunteers should apply to the honorary organiser of the Ulster Hospital Corps at 112 Beaufort Street, London, who will answer all inquiries. In addition an appeal is made to those who are willing to contribute hospital necessaries in the shape of stores, clothing, or hospital articles generally. It should be added that guarantees of funds against the event of mobilisation will be gratefully received by the committee. It is hardly necessary to state that no medical man, and still more no nurse, desirous to volunteer should do so until they have satisfied themselves of the duties expected of them, and the conditions of service, by making application to the authoritative source.

### OUR HOSPITAL SUNDAY APPEAL NUMBER.

Hospital Sunday falls this year on June 14, and, as usual, we publish this week, for distribution to all the churches of the Metropolis on the Sunday preceding the annual Sunday Fund appeal, a Special Number of The Hospital. To the outline of its articles which we published last week we are now able to add a full *résumé* of its contents. First of all the Chief Rabbi, Dr. Joseph Hertz, discusses in a special interview "The Inner Meaning of Hospital Sunday," and embodies in it a message to the congregations of London which members of all denominations who attend a place of worship on that day may well take to heart. As Hospital Sunday owes its origin to the clergy themselves, who are, therefore, more intimately associated with and responsible for the success of the collection than is the case with any other movement which they voice, the Special Appeal Number also contains an article on "The Churches and Hospital Sunday" in the form of a Lay Sermon to the London Clergy, which sets out their relation to this appeal by explaining the element of personal service by those in health for those who are ill, that really constitutes the embodiment in workaday life and work of one of the chief tenets of Christian life and doctrine. These articles on the religious side of the Hospital Sunday Fund movement are further supplemented by a financial article setting out the Position and Prospects of the Sunday Fund at the present time; and by a detailed and pictorial treatment of the work done by the London hospitals, both general and special, for every class of patient; and by an expert summary of the individual needs of each separate institution. In this way the clergy and members of the congregations with this Special Appeal Number in their hands not only will be brought to realise the meaning of their responsibility for the success of the Fund, but will also have the means of deciding which hospital appeals most to their sympathies. That the lighter minds and less responsible readers may also have their tastes and sympathies aroused there is also an illustrated article describing, with photographs, the work of "Sailor," the famous hospital dog collector of Bristol, who has collected so much cash for and received so many signs of appreciation from the Bristol Royal Infirmary. Members of the general public and all interested to obtain copies of this unique collection of articles on the Sunday Fund movement can receive copies of the Appeal Number, price one penny, on application to The Manager, The Scientific Press, Limited, 28 and 29 Southampton Street, Strand.

### NATIONAL MEDICAL UNION DINNER.

As reported in last week's issue of The Hospital, it has been decided to hold a dinner after the general meeting of the Union on Saturday, June 27. The dinner will take place that evening at 7.30 p.m., at the Café Monico, Piccadilly Circus, London, W. It is hoped that there will be a large attendance of members of the Union, and that they will take the opportunity of bringing guests with them, who, though not at present members, are interested in the Union and in sympathy with its aims and objects. Tickets for members and guests, price 7s. 6d. each, exclusive of wine, may be obtained from the Hon. Secretary of the Dinner Committee, National Medical Union Offices, 346 Strand, W.C. The work of the committee will be greatly helped if members will apply *as soon as possible* for tickets for themselves and for any guests they desire to bring, enclosing cheques or postal orders with their letters of application. The committee also would be particularly glad to hear from any members able and willing to promote the enjoyment of the evening by giving a song or recitation after the dinner. To meet the convenience of any members who may be pressed for time, we are asked to state that evening dress at the dinner will be optional.

### PRINCE ALEXANDER ON COMMITTEE WORK.

In offering the congratulations of the Middlesex Hospital to Prince Alexander at the quarterly Court last week on his appointment, as their President, to the post of Governor-General of Canada, the speakers made references to his work, the happiest of which was put forward by Sir James Kingston Fowler on behalf of the honorary staff. "We are indebted to Prince Alexander," he said, "for the way in which he has maintained the reputation of his House in regard to public honorary services in general and to this hospital in particular." This, indeed, is the point which gives sincerity to the congratulations of the governors. In the course of his reply Prince Alexander said: "The flattering references to my work in connection with the hospital are, I fear, not altogether deserved. It is true that I am keenly interested in it; " but he continued, after reference to the assistance afforded to him, " in work of this kind unsupported effort is almost certain to prove a failure." He added that his close association with the hospital had taught him much, and shown him the importance of the work in a fashion which less intimate acquaintance could not have done. " When I come back," the Prince concluded, " I will begin once more, and with you again go forward." This surely is no bad tribute to the genuine interest of hospital committee work to those who have brains enough to keep clearly in view the end for which the detail and routine are inevitable. These remarks should prove an inspiration, as they are a confession of faith, to hospital committeemen everywhere, who, we hope, will learn the lesson and draw the moral which Prince Alexander of Teck has done. The suggestion of Mrs. Turnbull, by the way, that a Canadian Ward should be established at Middlesex Hospital, is one worth consideration.

### THE LOST LINER: MEDICAL OFFICER'S STORY.

The most pregnant story of the marine disaster in the St. Lawrence which has given such an unexpected sombreness to many homes this Whitsuntide was told by Dr. F. J. Grant, who graduated in 1913 at McGill University. We left Quebec, he told a *Times* representative, on May 29, and early the next morning the collision occurred. The *Empress of Ireland*, which had some 1,500 passengers and crew on board, sighted the collier *Storstad*, and the liner's commander, Captain Kendall, signalled " I am continuing my course "; as the collier continued to approach the *Empress of Ireland* stopped, and then went full steam astern, but could not avoid the smaller vessel which rammed her amidships, and ripped open almost the whole of one side. She listed, filled, and went down in seventeen minutes. " There was no disorder," said Dr. Grant, " among the mass of the people on deck, and scores never left their bunks. On the *Storstad* and the *Lady Evelyn*, which was summoned by wireless, everything was done for the survivors. In five cases, however, the shock was too severe. In each case I was called, but the unfortunates died before anything could be done for them. Their last spark of energy had been exhausted. One other woman died as she was being taken ashore. I was rolled out of my berth by the list of the boat. I could not find the door. I could hear screams, but did not know what was wrong. Finally I managed to get out of my state room, but was unable to walk up the alley way because of the list of the boat. I scrambled along a wall and grasped a port-hole. I got my head out, and what was my astonishment to find the side of the vessel crowded with people. I called; someone reached down as I was trying to get my shoulders through the opening. This man pulled me out, and I, too, stood with them for a moment. The ship was pulled from under us, and we were all struggling in the water. The fog then lifted as if it had done its purpose. I could see about a mile away the lights of the steamer that I afterwards learned had struck us. I swam to it, and was picked up by a lifeboat that had just been launched. In it I returned to where the ship had gone down and helped to pick up those who were struggling in the water." Many tributes are paid to Dr. Grant for his aid to those who needed treatment, not only to those who were injured, but to those also who suffered from mental shock as a result of their terrible experiences. Here, again, we can all rejoice that even in a disaster such as this—the loss of life was over 1,000, and only 425 were saved—the hospital world could provide the man, but let us add the training, who and which were able to do all that skill and discipline could accomplish among the injured and exhausted few who survived the sinking of the liner.

### RETIREMENT OF A LIVERPOOL SURGEON.

It is announced that Mr. R. W. Murray, senior surgeon to the David Lewis Northern Hospital, Liverpool, is retiring from that institution and also from active professional practice. Mr. Murray is a Guy's man, who has spent nearly the whole of his surgical career in Liverpool. He has been surgeon to the Children's Infirmary there, as well as to the institution from which he now retires. Mr. Murray has written a great deal, more especially upon hare-lip and on hernia. In respect of the latter, he has for years stoutly maintained the view, now generally accepted as a result of his able advocacy, that hernial sacs are usually preformed (congenital), not developed by a gradual bulging in front of the hernial protrusion; a few years ago the latter view was that held and taught by almost all surgeons, but Mr. Murray's writings have brought about a change of surgical opinion on the point. Mr. Murray has long been noted for the high quality of his addresses on surgical subjects at the Medical Institution; and it is understood that he proposes to devote part of his well earned leisure to literary pursuits, as well as to travel.

### THE CLEANSING OF A VERMINOUS BUILDING.

Some remarkable disclosures have been made in a controversy between the Lambeth Board of Guardians and the Lambeth Borough Council. Some ten years ago, when the smallpox scare was at its height, a temporary building was erected, at the suggestion of the medical officer of health, near the Lambeth Infirmary, for the observation of suspected cases. The building was never used, but the Guardians placed in it the furniture and bedding of people who had to come into their institutions. A few months ago it was suggested that it might accommodate tuberculosis patients, but on examination it was found that the building and a great deal of the furniture stored there were in a verminous condition. The Clerk to the Guardians wrote asking for the advice of the medical officer, who promptly replied that the best way to deal with it was for the Guardians to buy a spray at a cost of about £2, and to spray the furniture with it. The suggestion that the Guardians should do the work themselves did not meet with their approbation, and consequently there has been considerable friction between the two bodies. Eventually the sanitary staff of the Borough carried out the work of disinfecting. The Council was naturally indignant that another public authority should have allowed any building to have got into such a condition, and it is to be hoped, for the credit of the Guardians, that in future they will keep a keener watch on those buildings where they store furniture and bedding of this description. Who shall see to the sanitation of the sanitary authorities?

### A NEW DEVELOPMENT OF THE PAY-WARD.

An interesting development has taken place in connection with the South London Hospital for Women, now being built near by Clapham Common, in the form of a League of professional and business women which has been started to endow or support one or more beds in the institution's private wards for the use of members. The League, it is hoped, will appeal to those genuine free-lances who live in flats, rooms, or residential clubs, and those who become members would have the privilege of free private treatment, or, according to their circumstances, at a reduction. Till the new hospital is opened, which, if hopes and contracts be fulfilled, will be the case in the spring of next year, the hospital's medical staff have conceded to the League free treatment, whether surgical or medical, at nursing homes approved by them. The hon. secretary is Miss Costello, 29 Bramham Gardens, S.W. This appears to be a development of the pay-ward and the maintained bed, the working of which will be watched with interest. The pay-ward for those who cannot afford the fees of a nursing home we all know, and the bed maintained by one class of workers for their own members is equally familiar; but this plan of the League appears to differ from both in that the beds which they hope to support or maintain are to be in the private wards of the hospital, and are therefore provided rather at reduced special fees than as beds maintained by subscription.

### THE PAY-WARD MOVEMENT IN JERSEY.

Our advocacy in recent years of the value, indeed necessity, of pay-wards in the voluntary hospitals for those who cannot afford the fees of nursing homes, into competition with which there is no necessity for hospital pay-wards to enter, has had many practical illustrations of late. We record above in these Notes the modification of pay-wards now suggested at the South London Hospital for Women, and the latest example comes from Jersey. There the Jersey General Dispensary and Infirmary, which is to be housed in a new institution, particulars of which will appear in our list of "Buildings Contemplated and in Progress" next week, is attempting to combine the charitable part of the work with contributory payments at half-a-guinea a week, and also with paying patients in private wards at not less than two guineas a week. Experience seems to show that on the whole this sum represents the wise figure. This hospital, by the way, with nice precision, draws a distinction between contributory patients and those called so loosely paying patients, pointing out that the former is the more exact term. Owing to the delays which have resulted from all negotiations with the Westaway Trustees, new arrangements for a site have been decided on. The movement for pay-wards is making slow but sure progress in Jersey.

### WOMEN'S HOSPITAL BURNT BY SUFFRAGETTES

It has been decided to double the present accommodation for children in the Dundee Royal Infirmary by allotting a second ward of thirty cots for this purpose. This does not mean that the accommodation for adults will be correspondingly restricted; and it is also stated that the arrangement is not regarded as either final or adequate, but merely the best that can be made for the time being until such time as Dundee is moved to provide itself with a children's hospital. The new ward will be ready in the autumn, when the new nurses' home will also be completed. The new women's hospital in this city, which was almost finished, and would have been open by the time these lines appear in print, has been set on fire by militant suffragettes and partially destroyed. The damage is estimated at £2,000, and is fortunately covered by insurance. Six months will be required for making good the damage. Insensate as the acts of militant suffragettes usually are, this seems to be a more than ordinarily insane exploit. We presume the idea of it is that women who do not want votes, or will not make themselves "militants" to get them, shall not have hospital accommodation either.

### THE TENDENCY OF TUBERCULOSIS APPOINTMENTS.

Among tuberculosis appointments up to date is that of Mr. Frederick J. C. Blackmore, M.R.C.S., L.R.C.P., to the post of chief medical officer to the Tuberculosis Dispensary, Woolwich. Mr. Blackmore, who qualified in 1909, for the past few years has been senior assistant medical officer at the Tuberculosis Centre, Birmingham, holding a resident appointment at the City of Birmingham Sanatorium. His publications include articles on the subject of tuberculosis in the medical papers, among which we may mention one upon the subject of "Tuberculosis Dispensaries in Theory and Practice," which he contributed to The Hospital of December 13 last. We hope we may note a very desirable tendency to fill tuberculosis dispensary and similar appointments with men who have had practical experience, clinical and administrative, of phthisical patients and institutions. Certainly the speed with which the so-called "crusade" led to the mushroom growth of appointments all over the country was accompanied at first by the very loosest demands on the part of the authorities for special qualifications of any kind.

### THE COMING OF AGE OF MEDICAL MISSIONS.

On Saturday, June 13, Commemoration Day, which celebrates the twenty-first year since the foundation of Livingstone College, Leyton, will take place at the institution. The occasion has peculiar interest for several reasons. It marks, in the first place, a definite step towards the goal when every mission centre in the East or among uncivilised and remote peoples shall have at least one fully qualified medical missioner, and it also shows the growing importance attached to the undoubted need that all missionaries should undergo at the very least a period of study in the elements of medicine and surgery. Livingstone College stands as the centre from which these two vital principles emanate as a factory for medical missionaries, and all who realise their fundamental importance will be glad to have an opportunity of supporting this still neglected but most interesting aspect of medical education and institutional practice. Moreover, the Principal of the College, Dr. Harford, will act as host for the last time, and that will give a weight to his words and a pregnancy to any views he may express which no other occasion could rival.

### THE FUTURE OF LIVINGSTONE COLLEGE.

Among several addresses under the presidency of the Bishop of Chelmsford, a member of the Mengo Mission Hospital in Uganda will speak from his experience in Central Africa and the East on the need of elementary medical training of missionaries, and other speakers will recount their experiences in the light of this need in such remote and distant places as Blackhead Island in the Arctic Circle, among the Eskimos, Tanganyika in Central Africa, and Inland China. But the serious business of the occasion will not stop here. Last year an appeal for £10,000 was issued to provide a centenary fund, of which £3,500 is required to pay off a mortgage, and of which the remainder would mainly be required to provide the nucleus of an endowment. So far only £640 has been subscribed, Many appreciate the value of the College, and also the work which Dr. Harford himself has done; it rests with these, therefore, to transfer their knowledge and appreciation to the general public, to make all who attend on Saturday, June 13, realise that the occasion is one of public importance, and, finally, by guaranteeing at least the £3,500 urgently required to complete Dr. Harford's work for him and ease his mind and responsibility by putting the finance of the College in order for the benefit of the new Principal, Dr. Loftus E. Wigram, who succeeds, as Vice-Principal under Dr. Harford, to the senior post.

### THE NEW SUPERINTENDENT AT BENENDEN SANATORIUM.

The paragraph which we published last week on the staff changes at the National Sanatorium, Benenden, Kent, is now out of date in the sense that further, and this time presumably permanent, appointments have been made. The new medical superintendent is Dr. William J. Cox, who succeeds Dr. W. Scarisbrick in this post. The new assistant medical officer is Dr. W. Wrigley Stacey, who succeeds Dr. A. Leitch. Dr. Cox, the medical superintendent, who is an M.B., Ch.B. of Victoria University, and holds the D.P.H. of Manchester also, was formerly medical superintendent of Mount Vernon Hospital for Consumption, Hampstead, and has also held the posts of resident medical officer of Crumpsall Workhouse Infirmary and house physician at Manchester Royal Infirmary. Dr. W. Wrigley Stacey is also a graduate of Victoria University, Manchester, where he took the degree of M.B.Ch.B. in 1910. These new appointments will be welcomed on the ground that nothing is more unsatisfactory to any institution than frequent changes in the staff, and we hope that they will prove permanent on that account.

### THE SALARIES OF POOR-LAW OFFICERS.

At the last meeting of the Lambeth Board of Guardians the infirmary committee reported that no application for the appointment of sixth assistant medical officer had been received in answer to the advertisement. The report admitted that this was due to the low salary offered—namely, £120 per annum, with the usual residential allowances. Originally the Guardians, to their credit, suggested that the salary should be £150, but the Local Government Board ruled this out and laid down £120. The committee recommended, and the Board agreed, that the circumstances should be communicated to the Local Government Board, with another appeal to be allowed to offer £150. It is extraordinary that while evidence is accumulating that medical men are able and determined to reject ill-paid posts, the Local Government Board, which ought, as trustee for the efficiency of the Poor-Law service, to be the first to realise that the adequate payment and recruiting of staffs is the surest road in the end to economy, is often found standing in the way of progressive Boards of Guardians, whom it is their duty to encourage and abet.

### MOTOR-CAR COTS.

The Nelson Hospital certainly receives its share of motor-accident cases. On Derby Day, we learn, three men were brought in with smashed heads. One succumbed to his injuries within two hours: regarding the second, the surgeon had never seen so severe a fracture. Within a month five children were received suffering from motor-car injuries. The demands upon the hospital continue to grow. Originally intended for twenty-four patients, within a few weeks of opening two more beds were installed. The accommodation has just been increased to twenty-eight beds and cots; but even this does not suffice. To provide further beds, structural alteration will be required. Why is not a proposal made to endow or maintain a "motor-car cot"? There is, it seems, a special need for this class of case, and were the suggestion brought before as many of the owners of motor-cars as the hospital could get in touch with, it might appeal to their sportsmanlike instincts.

At any rate, we throw out the suggestion to Mr. D. D. Kirkaldy and his committee for what it is worth. What are their views, and those of our readers?

### THE GENERAL MEDICAL COUNCIL AND DEATH CERTIFICATION.

The correspondents who have recently criticised in our columns (May 23, p. 214, and May 30, p. 241) the present defective methods of death registration and certification in this country will, doubtless, have noted that at the recent session of the General Medical Council the President dealt with this matter in his address. Sir Donald MacAlister stated that a memorandum setting forth relevant extracts from the Council's minutes was prepared in the office and sent to the present Registrar-General for his information. In acknowledging its receipt the Registrar-General informed the Council that fresh instructions were being issued which would effect certain improvements. In particular, local registrars are to be instructed that any death for which no certificate of the cause of death is produced from a registered practitioner must be reported to the Coroner; and, further, that the expression "medical certificate of the cause of death" means exclusively the certificate of a registered medical practitioner. This reform will do some good, though it is only one among many which are overdue. Up to the present the local registrar has exercised his own discretion whether or not he shall accept the certificate of an unregistered practitioner or refer the case to the Coroner for instructions. Many registrars habitually accept such certificates in large numbers, a practice which makes a dead letter of the whole object of death registration as an element in national hygiene. It is satisfactory that this option is now taken away from them, since most of them are qualified neither by education nor by experience to exercise it intelligently. Sir Donald MacAlister further stated that legislation will probably be required to effect other needful improvements in registration practice, and that the Executive Committee have the matter under consideration. We shall publish further correspondence next week.

### SCOTLAND'S FIRST HOMŒOPATHIC HOSPITAL.

On Thursday last week the Houldsworth Homœopathic Hospital, Glasgow, which is, writes a correspondent, the first institution of its kind in Scotland, was opened by Mrs. Paul Rottenburg. It owes its being largely to Miss Jane Houldsworth, of Ayr, and her sister, who gave £7,000 in memory of their brothers, both of whom were generous supporters of homœopathy, and Dr. R. Gibson Miller, of Glasgow. Five years ago a dispensary for the homœopathic treatment of the poor was started in the city, and it was to meet the increasing demands upon its resources and to demonstrate the benefits of the system that it was decided to purchase and equip premises for hospital purposes. Whilst the principal object is the treatment of the poor, it is intended to reserve a few rooms for those who are unable to pay for treatment in a nursing home. Miss Elizabeth A. Hay, formerly on the nursing staff of the Glasgow Western Infirmary, has been appointed matron. After the opening Dr. C. E. Wheeler, representing the British Homœopathic Association, delivered a lecture on "Homœopathy after a Hundred Years," in which he traced the life and work of Hahnemann and the development and present position of homœopathy.

### A PICTURE THEATRE AS A HOSPITAL NUISANCE

Is a picture theatre situated within a few yards of a hospital a nuisance? was a question raised at a recent meeting of the London County Council. Mr. Yeo, M.P., asked the chairman of the Theatres and Music-Halls Committee whether it was a fact that it was proposed to convert the disused school premises in Brunswick Road, Poplar, into a cinematograph theatre, and, if so, whether the Committee, when dealing with the application for the licence, would consider the serious annoyance to patients at Poplar Hospital which would be caused by the proximity of a cinematograph theatre. Mr. Herbert Lidiard replied that the answer to the first question was in the affirmative, but, though a notice of the intention to erect a cinematograph theatre was exhibited on the site before the plans were considered by the Committee, no representations on the subject were made by the hospital authorities. He was sure the Committee would see the representatives of the hospital with very much pleasure indeed if they wished to explain the position. What are our readers' views?

### INFIRMARY SUPERINTENDENT AS SANITARY INSPECTOR.

Dr. H. W. Bruce, the medical superintendent of the Southwark Infirmary, has replied in a report to the Guardians to the suggestion made by the Camberwell Borough Council that the nuisance at the infirmary, due to the close proximity of a refuse siding, was merely an æsthetic one. Dr. Bruce states that since his report last June, with the exception of a heap of refuse left standing for two days, he had not observed any large accumulations on the siding. The surface of the siding, which was considerable in area, is of earth, and it is strewn all over with refuse which has dropped or been blown from the trucks, and with manure. It cannot be cleansed. It is a condition of things, he adds, which no private individual would be allowed to maintain, and he asserted again that it is a disgrace to those responsible for it. Dr. Stevens, the medical officer of health, who, with Dr. Cautley, of the Belgrave Hospital for Children, has visited the infirmary, recommended that the milk given to the children should be boiled and not pasteurised, but Dr. Bruce remarks that the boiling of milk was condemned by every authority of repute on the care of children. Dr. Cautley himself has written "Pasteurisation is an efficient process for destroying the microbes in milk." The milk was boiled at the infirmary until some years ago, when, in agreement with the consensus among medical men, Dr. Bruce initiated the process of pasteurisation followed by rapid cooling.

### SOUTHWARK GUARDIANS APPEAL TO HEADQUARTERS.

Dr. Bruce, in conclusion, says that the failure of the investigators to find anything in the state of the infirmary to which the outbreak of enteritis could be attributed strengthened his conviction that the refuse gives rise to disease. He considered that the gist of the controversy could be summed up in two questions: Is dustbin refuse a possible cause of disease? and Can dust be blown 100 yards? If the answers to these questions were in the affirmative, and they must be, then he said that the use of this siding for the shooting of refuse ought to be discontinued. He deplored the attitude of Dr. Stevens towards these infantile deaths. The Southwark Board of Guardians were in thorough accord with Dr. Bruce, and decided to send a copy of his report to the Local Government Board, with a request that immediate steps be taken to compel the Camberwell Borough Council to remove the nuisance complained of.

### RIFF PIRATES AND LONDON HOSPITALS.

At first glance it would be difficult to understand what possible connection there could be between Mediterranean pirate galleys and London voluntary hospitals. Nevertheless there is a connecting link, distant and roundabout though it may be. The existence of this link is recalled by the recent announcement that donations to various hospitals have been made by the trustees of Smith's Charity: namely, £500 to the Hospital for Consumption and Diseases of the Chest, Brompton; £250 to the Prince of Wales's General Hospital, Tottenham; £500 to the Chelsea Hospital for Women towards the rebuilding fund, and £100 towards the same institution's convalescent home at St. Leonards; and £200 to Queen Charlotte's Hospital. Smith's Charity was originally founded and endowed, we believe, to provide a fund for ransoming London sailors from captivity among the Moors. This was, of course, at a time when the Algerine, Moorish, and Tunisian pirates swept the Mediterranean in their galleys, and carried off the crews of hapless merchantmen into slavery. Nowadays the funds are not needed for the pious testator's intentions, so the trustees put them to very excellent use by contributing liberally to hospitals. The Brompton and the Chelsea Hospitals have especially strong claims on the trustees in that they adjoin the estate which provides the income of the trust.

### DEATH OF AN ARDENT NON-PANELLER: JAMES SMITH.

The death took place on May 28 of Dr. James Smith, the well-known Edinburgh practitioner, and an active member of the National Medical Union. It is impossible to talk of Dr. James Smith without referring to his attitude and action during the turmoil which preceded and followed the advent of the Insurance Act in Edinburgh. "A Liberal and a Radical in politics, with strong altruistic sentiments," writes one who knew him well, "everything that concerned genuine Socialism was for him a fascination and delight, and he busied himself to the full in many schemes of social reform. But as a doctor he always recognised the limitations of all human efforts in this direction, and for some time past many of the so-called socialistic projects which were being planned caused him great and increasing misgiving. The appearance of the Insurance Act brought matters to a crisis for him. Realising that the healing art, like religion, must lose its identity when it is sought to be inculcated by law and police, he detected in it the beginning of unending harm to the community. Recognising in that Act the violation of divine wisdom and law to 'render unto Cæsar that which is Cæsar's and unto God that which is God's,' its ungodliness shocked him, its disregard for the teachings of history astounded him, and its assumption of the highest benevolence combined with its 9d. for 4d. appeal to the lowest of human feelings excited in him anger and contempt fierce and scathing. In spite of the fact that on more than one occasion his lay friends, alarmed about his health and concerned about his prospects, begged him to put his feelings aside and sign on to the panel, he resolutely refused, and though it would not be true to say that this refusal meant for him direct pecuniary loss, there is no doubt that the wear and tear in that anxious year for medical Edinburgh was the factor which broke down beyond repair his never very robust physical health. His spirit, however, remained indomitable." He graduated M.B., C.M. at Edinburgh in 1888, and M.D. at the same University in 1894. Dr. James Smith was a member of the National Medical Union, and a strong believer in its cause. His life's work was spent chiefly among the working classes, and when the Insurance Act was inaugurated he stood to lose considerably. He refused, however, to touch it, and it is significant that he suffered, we learn, no financial loss by his courage. He was always in the front during the fight in Edinburgh

### THE WHITSUNTIDE DRUG MARKET.

The dullness in the drug market has been accentuated by the Whitsuntide holidays, but it is not unlikely that an improvement will set in shortly. There is little change in the position of opium. The value of glycerine is well maintained, and no decline is anticipated. Formaldehyde is lower. Cloves are unchanged in price, but an advance is considered probable. There is practically no alteration in quotations for cod-liver oil; the yield of oil from the Finmarken fishery has been about half that of last year, but there is a large yield from the fishings in other districts. Menthol tends lower in price, and the demand is very quiet. Camphor is unchanged since the recent advance, but a further rise is not altogether improbable. Cocaine has a firmer tone and a small advance in price may possibly take place, but no substantial rise is looked for. Essence of lemon still tends downwards in price. Thymol is dearer. The price of buchu leaves has an upward tendency.

